# Creating national care standards for neonatal intensive care units in 2007

**Published:** 2010

**Authors:** Mehri Golchin, Hayedeh Heidari, Shohreh Ziaie, Shayesteh Salehi

**Affiliations:** * Department of Pediatric Nursing, School of Nursing and Midwifery, Isfahan University of Medical Sciences, Isfahan, Iran; ** Student, School of Nursing and Midwifery, Isfahan University of Medical Sciences, Isfahan, Iran; *** Educational Management, School of Nursing and Midwifery, Islamic Azad University, Khorasgan branch, Isfahan, Iran

**Keywords:** Newborns, standards, intensive care unit

## Abstract

**BACKGROUND:**

Infant mortality rate was reported 3.18 in 1000 births in Iran. International organizations such as World Health Organization (WHO) and United Nations Children’s Fund (UNICEF) consider applicable standards essential for providing effective health services in hospitals and health centers. Therefore, it is essential to create national care standards for neonatal intensive care units (NICU) in Iran.

**METHODS:**

This was a multiple triangulation study conducted in 2007. In the first step, international standards were extracted from appropriate sites. Then, using Delphi method, as well as the viewpoints of 15 experts in clinical medical sciences, a set of suggested standards for intensive care unit was prepared. In the third step, 42 clinical science experts of Iran were selected, and their viewpoints on applicability of the suggested standards were investigated through a descriptive survey method. Data obtained in this step were analyzed using descriptive statistics.

**RESULTS:**

First, intensive care standards were extracted; then clinical science experts reviewed the suitability and applicability of suggested set of standards for Iran and finalized them. Finally, 386 standards for intensive care were drafted and approved by 77.5% to 100% desirability rate for NICUs of Iran.

**CONCLUSIONS:**

The findings of the study showed that most standards were either appropriate or fairly appropriate. So, necessary changes in final standards were made based on subjects, viewpoints and suggestions as well as the results of consulting with supervisors.

Giving importance to health care would have obvious effects on the improvement of regional health and existing economic resources. Nowadays, development of technologies and changes in service needs have increased the request for inservice educations. Efforts to improve supervision and evaluation through appropriate organization can lead to creation of standards to assess performance. Standards include regulations, guidelines and features of activities or results and are created to be used in specific profession through consensus of experts in that profession. In nursing profession, the essential factor for managing nursing services is evaluation systems, which means following standards of performance, goals, planning operational programs, quantity and quality of health care services, working hours and costs.^1^,^2^ Recent development in science and technologies has improved the quality of nursing programs and as a result, nurses have faced advanced techniques and professional skills. Also, nurses cooperate with other health care team members as coordinators. Therefore, evaluating quality of nursing care is very important.^3^ Nursing profession needs laws and regulations to provide the same care that good nurses provide within similar situations. As a result, providing high quality care should be based on determined standards.^4^ Developing neonatal care standards and following them, especially those who need intensive care have decreased infant mortality. For example, in the United States the infant mortality rate reduced from 10 in 1000 births in 1987 to 6.9 in 2000.^5^

The goal of standards is to create quality levels and desirable services to protect the society by nursing care facilities. Standards show the society that nurses are directly responsible for the quality of nursing services and adjust the quality level of their services to reach the desirable level.^6^

Since the infant mortality in Iran is reported 3.18 in 1000 births,^7^ which is much higher than this rate in the developed countries, it is essential to create health care standards for Iran to reduce the long term problems facing infants with high risk and to remove functions of taste among infant nurses. International organizations such as World Health Organization (WHO) and United Nations Children’s Fund (UNICEF) consider applicable standards essential for providing effective health services in hospitals and health centers and neonatal care standards, which are components of children’s rights, can prevent infant mortality.^8^

Since standards for neonatal intensive care units in Iran have not been created and the authorities have felt the necessity of standards for NICUs more than ever and the researchers also have come to the importance of them in their clinical experiences, this study is conducted to develop national care standards for neonatal intensive care units in Iran based on the international standards of 2006. The special aims of the study included determining criteria for admission, care during hospitalization, infection control and hospital policies and discharge criteria for NICUs.

## Methods

This study was based on multiple triangulation approach. Sampling was purposive and data were collected by a questionnaire, which included two sections of demographic data and standards. Answering was based on a 3 Likert scale of suitable, relatively suitable and unsuitable. Study population included experts of nursing and medical sciences. Entry criteria included having a master degree in nursing or be working for at least 2 years in NICU for nurses with undergraduate degree, having pediatric specialty for doctors, be willing to participate in the study. The option to leave the study at any time was open. Reliability and validity of the questionnaire was predicted using professionals ideas and consensus.^9^

The study was conducted in three steps. In the first step, the standards of neonatal care of 10 countries and states were extracted from credible sources via internet, databases and other texts and the questionnaire items were developed using these standards. In the second step, Delphi technique (classic) was used to assess the reliability of the questionnaire. In order to use Delphi technique, the sample in the second step included 15 experts who had the entry criteria. They were 5 nursing faculty members (specialized in infants), one midwifery faculty member (specialized in mother and infant), 5 pediatrics who were faculty members of the Isfahan University of Medical Sciences and 3 nurses with master degree or bachelor degree with working experiences in NICU. After the questionnaires were completed, the viewpoints of experts were considered for editing the first draft, which was given again to the 15 experts. Standards were accepted with a consensus of 90% and the final standards were created. Finally, in the third step of the study, a survey method with descriptive design was followed for the national poll on NICU standards. In this step, the questionnaire which was prepared in the second step was sent to 60 experts from nursing schools and hospitals who had the inclusion criteria. The number 60 was considered by calculating the probable withdrawal of samples.

In the third step of the study, the researcher traveled to different cities including Isfahan, Ahvaz, Tehran (health centers of Shahid Beheshti University, Tehran University and Iran University), Shiraz, Tabriz, Eurmieh and Yazd to deliver the questionnaire to experts with inclusion criteria in these places and gave them enough time (two weeks to one month) to complete the questionnaire and followed up with a second trip to collect completed questionnaires. In total, 42 experts completed the questionnaires (30% did not complete and return the questionnaire). After revising, the national standards in accordance with executive, cultural, social and economic situations of Iran were prepared with a consensus of higher than 70%.

Variables of the two sections of the questionnaire were analyzed using SPSS and frequency distribution.

## Results

Most participants in the study (40.5%) had a master degree, were in the age range of 36-45 (45.2%) and had 2 to 10 years of work experience (52.34%) and 64.82% of participants had 6 to 10 years of work experience in ICU.

By extracting care standards in different steps of the study, 386 standards for NICU in Iran were created (Table 1). Most of these standards were favorable and relatively favorable (95% to 100%), but some of them that were less favorable (70% to 94%) included standards of the first study objective: in the “admission criteria”, level II or intermediate admission, hospitalization of infants with APGAR score of 4-6 in the fifth minute was 82.5% favorable and hospi talization of infants with a small surgery in past 24 hours was 85% favorable. In the level III or intensive admission, hospitalization of infants with life threatening congenital defects was 87.5% favorable. In nurse duty at the admission time, providing care plan within 24 hours from the time of admission was 92.5% favorable. Less favorable standards related to the second objective of the study, “care during hospitalization” included: diagnosis and treatment of apnea 92.5%, educating parents for taking care of their infants 92.5%, nurses’ responsibilities in providing prescribed care or transfusion of blood or blood products by two nurses (supervising nurse and responsible nurse) 92.5%, mouth wash every 2 hours after feeding infants receiving mechanical ventilation or NPO 80%, placing all infants under warmers or in the incubators 90%, daily weighing of infants with special or severe conditions 85%, physical check up for patients with special conditions such as lung hypertension every 12 hours 77.5%, monitoring growth process every week 90.5%, controlling and recording heart rate, respiration, oxygen saturation in NICU at least once per hour 90%, controlling peripheral blood pressure in NICU at least once per working shifts 92.5%, controlling temperature using underarm at the time of admission and then every 4 hours 90%, encouraging mothers to use electric breast pumps if necessary 90%, changing catheters every 3 days 87.5%, checking following items every 8 hours: bowel sounds, round belly size, intestinal torsion, existence of tachypneaapnea and brady cardia 87.5%, taking medicine within one hour before and after prescribed time 87.5%, recording and setting time for using transducer, caliber air in each working shift 92.5%, checking mouth and lips every 4 hours for possible harms and pressure due to used techniques 92.5%, setting arterial oxygen to maintain within 88 to 96 percent in infants 92.5%, using usual pain killers such as mothers’ nipple, breastfeeding and topical ointments (EMLA cream) 92.5%, bathing premature infants with sterile water 77.5%, giving pain killer or tranquilizers according to physicians’ prescription to reduce stress and energy consumption 87.5%, determining ABG (arterial blood gases) during admission and in case of respiratory changes with physicians’ prescription 87.5%, using glycerin in rectum if baby has no bowel movement for 24 hours 80%, washing skin just if it is necessary 90%, and comforting and using pain killer if the baby is restless 90%. Standards related to the third objective of the study, “infection control and hospital policies” included cooperation of the leader of medical team (pediatric with required criteria) and nursing manager in planning, executing, and controlling budget in the ward, which was favorable 92.5%. Standards of the fourth study objective, “discharge criteria” were all favorable 95% to 100%.

**Table 1:** Distribution of NICU standards based on study goals

Goals	Standards	n
1- Admission criteria	1-1- Admission in intermediate (level II)	14
	1-2- Admission in NICU (level III)	7
	1-3- Duty of nurse during admission	17
	1-4- Care givers be able to do the below stages	20
	1-5- Responsibility of nurse in care giving	17
	Total	75
2- Care during hospitalization	2-1- Routine care	23
	2-2- Weight	4
	2-3- Physical examination	6
	2-4- Vital signs	9
	2-5- Temperature regulation in premature neonate	38
	2-6- Intake and output	5
	2-7- Feeding:	-
	2-7-1- Oral	5
	2-7-2- Fluid therapy and IV	14
	2-7-3- Intestinal feeding (bowel feeding)	28
	2-8- Drug	4
	2-9- Respiratory failure in neonates	12
	2-10- Monitoring	7
	2-11- Homodynamic monitoring	7
	2-12- Rules of bradycardia and apnea	6
	2-13- Ventilator	27
	2-14- Oxygen therapy in those who are not connected to ventilator	11
	2-15- Pulse oximetry	8
	2-16- Care giving in NICU	16
	2-17- Phototherapy	15
	2-18- Skin care	22
	2-19- Exchange transfusion in neonate	13
	Total	280
3- Infection control and hospital policies	3-1- Infection control policies	10
	3-2- Hospital policies	16
	3-2-1- Ward transmission criteria for newborns	3
	Total	29
4- Discharge criteria	4-1- Criteria of discharge	5
	Total	5

## Discussion

Based on the results of the study, standards were revised based on suggestions and based on being favorable or not being favorable and the final version of national care standards were created. Standards that were 95 to 100 percent favorable were used as they were except for copyedit cases. Standards that were 70 to 90 percent favorable were revised based on the suggestions of participants and scholars and standards that were favorable less than 70% were omitted.

In general, one of the reasons for consensus less than 90% for some standards is lack of nurses in health centers which makes nurses not to be involved in the decision-making process and reviewing patients’ problems.

Regarding standards of number of human resources in intensive units, Aiken et al (2003) said that reducing number of nurses causes increase of mortality and morbidity. Also, 10% increase in number of nurses with a bachelor degree reduces 5% of mortality rate of patients.^10^ Seago et al in 2000 mentioned the increase of mortality risk for every extra patient in the wards.^11^ Needleman et al in 2000 said that the presence of professional staff in wards reduces the length of hospitalization and incidence of hospital infections.^12^ Some other important factors for nurses who work in ICU are skills and experience. Using skillful nurses reduces health care costs. In addition, Duke et al in 2000 mentioned the necessity of ICU standards to reduce mortality of infants and recruiting experienced staff for ICU to decrease the incidence of septicemia and pneumonia in babies.^13^ Considering the development of technologies in medical sciences, the necessity of recruiting professional nurses especially in ICUs are very obvious. Finally, by conducting a survey, final standards were prepared (Table 1). The final standards included 21 criteria of admission that are in accordance with standards of Chicago, England and Lebanon, 17 standards for nurses duties at the time of admission, which are in agreement with those of Chicago, 20 standards for abilities of health care personnel in following these standards, which are in agreement with those of Chicago and Lebanon, 17 standards of nurses duties in providing these cares, which were in accordance with those of Chicago, England and the United States, 280 standards of care during hospitalization which were in accordance with those in Chicago, North Carolina hospitals and nursing committee, 5 discharge criteria which were in accordance with American Academy of Pediatrics, 16 standards of hospital policies along with 3 standards related to transferring criteria which were in accordance with those of Lebanon and England and 10 standards of infection control policies which were in accordance with those of Nursing Committee of North Carolina.^14^

Most countries regardless of their wealth and size consider health and health services a major issue and most developing countries are trying to develop a health care system that can target the major needs of their societies.^10^ Therefore, extracting and developing national care standards for NICU in Iran can be used as guidelines for related organizations such as Ministry of Health, Deputy of Treatment and hospitals to solve neonatal problems and improve their health and in general improve the quality of health in Iran. It is recommended that related organizations take advantage of the results of this study^14^ to revise clinical nursing based on standards and to improve the quality of nursing performance and quality of health services in the hospitals all around the country.

The authors declare no conflict of interest in this study. Ethical committee approved the study.
